# Exploring the role of juvenile hormone and vitellogenin in reproduction and social behavior in bumble bees

**DOI:** 10.1186/1471-2148-14-45

**Published:** 2014-03-11

**Authors:** Etya Amsalem, Osnat Malka, Christina Grozinger, Abraham Hefetz

**Affiliations:** 1 Department of Entomology, Center for Pollinator Research, Center for Chemical Ecology, The Pennsylvania State University, University Park, PA 16802, USA; 2 Department of Zoology, George S. Wise Faculty of Life Sciences, Tel Aviv University, Tel Aviv, Israel

**Keywords:** Vitellogenin, Aggression, *Bombus terrestris*, Social insects, Reproduction

## Abstract

**Background:**

The genetic and physiological pathways regulating behavior in solitary species are hypothesized to have been co-opted to regulate social behavior in social species. One classic example is the interaction between vitellogenin (an egg-yolk and storage protein) and juvenile hormone, which are positively correlated in most insect species but have modified interactions in highly eusocial insects. In some of these species (including some termites, ants, and the honey bee), juvenile hormone and vitellogenin levels are negatively correlated and juvenile hormone has shifted its role from a gonadotropin to a regulator of maturation and division of labor in the primarily sterile workers. The function of vitellogenin also seems to have broadened to encompass similar roles. Thus, the functions and molecular interactions of juvenile hormone and vitellogenin are hypothesized to have undergone changes during the evolution of eusociality, but the mechanisms underlying these changes are unknown.

Bumble bees offer an excellent model system for testing how the relationship between juvenile hormone and vitellogenin evolved from solitary to social species. Bumble bee colonies are primitively eusocial and comprised of a single reproductive queen and facultatively sterile workers. In *Bombus terrestris*, juvenile hormone retains its ancestral role as a gonadotropin and is also hypothesized to regulate aggressive behavior. However, the function of vitellogenin and its interactions with juvenile hormone have not yet been characterized.

**Results:**

By characterizing vitellogenin RNA expression levels (*vg*) in *B. terrestris* we show that *vg* is not associated with task and only partially associated with worker age, queen presence, and caste (queen vs worker). The correlations of *vg* with ovarian activation were not consistent across experiments, but both *vg* and ovarian activation were significantly associated with levels of aggression experienced by workers. Treatment with juvenile hormone did not affect *vg* levels in queenless groups.

**Conclusions:**

We suggest that social interactions affect *vg* levels more strongly than a worker’s reproductive physiological state, and that juvenile hormone and *vg* are uncoupled in this species. Thus, although juvenile hormone maintains its traditional role as gonadotropin in *B. terrestris*, *vg* has already been co-opted into a novel role, consistent with the model that *Bombus* represents an intermediate stage in the evolution of eusociality.

## Background

The proximate and ultimate mechanisms leading to the evolution of eusocial behavior, in which there is a reproductive division of labor between highly fecund females (queens) and largely sterile females (workers), has long fascinated biologists. It has been hypothesized that eusocial behavior evolved from solitary behavior via changes in expression of existing “toolkits” of genes [1], in which ancient molecular and physiological pathways became modified and expressed in new contexts, thereby leading to profound behavioral and physiological changes. This is exemplified in the major functional changes that have occurred in juvenile hormone (JH), a principle regulator of physiological processes in insects, during the evolution of eusociality. However, except in the case of *Apis mellifera*, whether and how the downstream transcriptional responses to JH evolved have not been explored. Here, we examined the interactions between JH and one of its main transcriptional targets, vitellogenin (*vg*), in a primitively eusocial species (the bumble bee *Bombus terrestris*), and compare our results with studies of the interactions between JH and vitellogenin in other solitary and social species.

JH displays pleiotropic functions during the insect’s life cycle. In pre-adult stages it regulates developmental processes, while in adult females it induces vitellogenesis (the production of the major yolk protein) [2-4]. However, while the role of JH in development (including pre-adult caste differentiation) appears to be widely conserved across insect species, its role in reproduction has been subjected to many modifications. In some highly eusocial insects, such as the honey bee and several termite and ant species, it no longer acts as a gonadotropin but instead regulates maturation and the division of labor in the worker caste [2,5-12]. Modifications in the hormone’s traditional role have also been reported in several non-social insects in which JH is either not essential for female reproduction or is not an absolute requirement for ovarian activation [13-20]. Furthermore, in several primitively eusocial hymenopteran species JH regulates formation and maintenance of dominance hierarchies in addition to its gonadotropin role [21-23].

Many effects of JH appear to be mediated by vitellogenin, and JH and vitellogenin interact in many species [24-26]. As the major egg yolk protein in insects, vitellogenin is synthesized in the abdominal fat body, released into the hemolymph, and sequestered by the developing oocytes [24,25]. In most insects, mainly non-social species, JH positively regulates vitellogenin levels [18,20,27-34]. For example, in adult female *Locusta migratoria*, blocking JH action either by ablation of the corpora allata (the site of JH synthesis) or by treatment with JH inhibitors abolishes vitellogenin production. Furthermore, vitellogenin biosynthesis can be induced in these females by injection or topical application of JH or an active JH analogue [24,35]. An exception is the parasitic wasp *Pteromalus puparum*, a non-social Hymenopteran species in which *vg* expression and JH titers are negatively correlated, and JH seems to play only a secondary role in stimulating oocyte growth [13].

In contrast, social insect species exhibit a much more complex interplay between JH and vitellogenin, and vitellogenin appears to have acquired new functions. For example, in honey bee (*Apis mellifera*) workers, high JH levels significantly reduce *vg* transcription as well as vitellogenin protein levels [36-38]. Interestingly, in the honey bee, vitellogenin operates in a negative feedback loop with JH, apparently slowing the onset of foraging behavior by suppressing JH titers, and thus appears to function as more than just an egg-yolk protein [36,38-40]. Although in most of the best studied ant species, including *Solenopsis invicta*[41] and *Pogonomyrmex barbatus*[42], JH retains its gonadotropin function, in several primitively eusocial ant species JH levels and vitellogenin levels and/or egg production are negatively correlated: topical application of JH to founding queens reduced egg production in *Harpegnathos saltator*[43] and reduced fertility in *Streblognathus peetersi*[44]. In some termite species, JH does not always stimulate vitellogenesis in reproductives. For example, JH synthesis correlates with the number of vitellogenic ovarioles in nymphoids but not in ergatoids of *Reticulitermes flavipes*[6,7], and an elevated JH titer may inhibit reproductive processes in immature alates of *Zootermopsis angusticollis*[5]. In *Hodotermopsis sjostedti*, JH titers of secondary reproductives are low compared to nymphs, pre-soldiers, and soldiers [8,9]; whereas in queens of the termite *Reticulitemes speratus*, JH and vitellogenin were found to correlate in an offset pattern, with an increase in JH titer preceding the rise of *vg* expression levels [45]. Although the number of species studied is still limited, we can hypothesize that JH and vitellogenin have undergone a change in their roles and the way they interact during the transition from solitary to sociality [4].

*Bombus terrestris* colonies offer an excellent model system in which to examine how the interactions between JH and vitellogenin changed during the evolution of eusociality. *B. terrestris* are eusocial, with a reproductive division of labor between morphologically distinct castes [46], and JH has maintained its original role as gonadotropin and is positively correlated with reproduction in both queens and workers [22,47-49]. However, the role of vitellogenin and its interactions with JH have not been explored in this species, and whether vitellogenin retains its ancestral role and functions largely in reproduction or if it has acquired other functions remains elusive. Furthermore, the interactions between vitellogenin and JH are also uncharacterized in this species: it is unknown if these two factors follow the ancestral pattern (positively correlated), have become uncoupled, or are negatively correlated, as observed in other eusocial Hymenopteran species.

In many social insects (mainly primitively social species), reproductive competition between the queen and workers and among workers is accompanied by overt aggression. This is particularly apparent in *B. terrestris,* where aggression is a major means by which to establish reproductive dominance in queen-worker and worker-worker competition [46,50-56]. JH titers were hypothesized to correlate with dominance and aggressive behavior in bumblebee workers, but the experimental results of several studies were equivocal [22,49]. Aggression also precedes ovarian activation in workers [54] and previous aggressive encounters by workers are sufficient to establish the reproductive hierarchy among them [56]. This raises an intriguing possibility that the molecular and physiological processes that regulate aggressive behavior (and therefore shape social context) occur upstream to the processes involved with vitellogenesis and ovarian activation. Most of the studies to date, to our knowledge, that sought to explore the relation between JH and vitellogenin were carried out with either non-social species or highly eusocial insects, in which aggression is rare or absent. In the bumble bee *B. terrestris*, aggression is a major factor in colony social behavior, but the impacts of dominance hierarchy on the interplay between JH and vitellogenin have not been examined.

Here, we investigated the interactions between JH and *vg* RNA expression levels. First, we examined *vg* expression specificity in relation to caste, reproduction, and worker task allocation, by monitoring its levels in virgin and mated queens, sterile and reproductive workers, and workers engaged in foraging or brood care. Second, since worker ovarian activation is associated with multiple factors (age, presence or absence of the queen, or position in the dominance hierarchy) we uncoupled all of these factors by manipulative experiments and monitored how these affect *vg* expression. We then extended this experiment by examining the effect of JH regulation on *vg* expression in workers introduced into two different kinds of social groups, in which they experienced different levels of aggression.

## Results

### Experiment 1: Examining the association of *vg* expression with caste, ovarian activation and worker task in *B. terrestris*

We measured ovarian activation and *vg* expression levels in the heads of queens and workers (Table 1) and statistically compared the results across the various groups (Table 2). Levels of *vg* were significantly higher (6x) in fertile queens than in 10 day-old, queenless (QL), fertile workers. Virgin queens (with inactivated ovaries) also had higher *vg* expression levels compared to 4-day-old, queenright (QR), sterile workers (2.4x), but this difference was not significant. Levels were also significantly higher (40x) in fertile queens compared to virgin queens, and significantly higher (16x) in 10-day-old, QL, fertile workers compared to 4-day-old, QR, sterile workers. Finally, although ovarian activation levels were significantly higher in nurses than in same-age foragers, *vg* levels were not significantly different.

**Table 1:** **Relative *vg *** **RNA expression levels in bumble bee queens and workers**

**Bee type**	**Ovarian status (mean oocyte size mm)**	***Vitellogenin *** **RNA expression levels**
Active queen (n=6)	3.2±0.02	165.6±70.2
Virgin queen (n=6)	0.09±0.009	4.3±2.7
Fertile workers (n=6)	2.52±0.06	29.6±12.1
Sterile workers (n=6)	0.06±0.006	1.81±0.56
Foragers (n=8)	0.11±0.04	4.08±1.1
Nurses (n=8)	0.64±0.34	5.81±2.1
5 days, QR (n=4)	0.18±0.04	3.6±1.3
8 days, QR (n=4)	0.23±0.05	11.3±7.7
5 days, QL (n=4)	0.77±0.08	16.4±6.6
8 days, QL (n=4)	2.48±0.22	16.9±5.6

Data are presented as means ± SE.

**Table 2:** **Statistical analysis of the factors influencing *vg *** **expression levels**

	**First group**	**Second group**	**Statistics**
Caste	Fertile queen (n=6)	Fertile workers (n=6)	**Ovaries: U=0, p=0.005; *vg*: U=4, p=0.03**
	Virgin queen (n=6)	Sterile workers (n=6)	**Ovaries: U=4.5, p=0.03; *vg*: U=16, p=0.8**
Reproduction	Fertile queens (n=6)	Virgin queen (n=6)	**Ovaries: U=0, p=0.005; *vg*: U=0, p=0.005**
	Fertile workers (n=6)	Sterile workers (n=6)	**Ovaries: U=0, p=0.005; *vg*: U=0, p=0.005**
Task	Foragers(n=8)	Nurses (n=8)	**Ovaries: T=2, p=0.025; *vg*: T=14, p=0.57**
Age	5 days, QR (n=4)	8 days, QR (n=4)	**Ovaries: U=7, p=0.88; *vg*: U=6, p=0.66**
	5 days, QL (n=4)	8 days, QL (n=4)	**Ovaries: U=0, p=0.03; *vg*: U=8, p=1**
Social condition	5 days, QR (n=4)	5 days, QL (n=4)	**Ovaries: U=0, p=0.03; *vg***: **U=6, p=0.06**
	8 days, QL (n=4)	8 days, QR (n=4)	**Ovaries: U=0, p=0.03; *vg*: U=1, p=0.66**

Comparisons of caste, reproduction, age and social condition were performed using a Mann-Whitney test. Task was compared using a Wilcoxon matched paired test.

Levels of *vg* thus seem to be affected by both caste and reproductive state, but not by task. However, there were many parameters that differed among our fertile and sterile workers in addition to ovarian activation, including age and social context (presence or absence of the queen). In the following experiment, we uncoupled the effects of age, social context (queen presence/absence), and ovarian activation, in order to determine which, if any, factor was primarily associated with *vg* expression levels.

### Experiment 2: Uncoupling the effects of age, ovarian activation and queen presence/absence on *vg* RNA expression levels in workers

The effects of age and queen presence/absence on ovarian activation and *vg* levels were examined in cages of 5- and 8-day-old workers that were kept either in QL or QR groups (Tables 1 and 2). QL workers usually posses ready-to-lay eggs at the age of ~7 days, thus only 8-day old workers were predicted to have vitellogenic oocytes in their ovaries. Among the QR workers, neither *vg* levels nor ovarian activation were significantly different between 5-day and 8-day old workers. Among the QL groups, 5-day-old workers had significantly lower ovarian activation, but still comparable levels of *vg* expression compared to the 8-day-old workers.

In this same group of workers, we examined the effects of social condition (QL vs. QR) on ovarian activation and *vg* levels. Both 5- and 8-day-old workers in QL groups had significantly higher ovarian activation than age-matched workers in QR groups, but levels of *vg* were again not significantly different.

In these experiments, we examined the expression of *vg* in the dominant worker (as determined by ovarian activation level) in each group (see Methods). If *vg* levels are associated with dominance, this may explain the lack of variation across the groups. Thus, we further explored the effects of social interactions on *vg* expression in QL groups of workers.

### Experiment 3: Examining the impact of social interactions (aggression) on *vg* RNA expression levels in queenless worker groups

In QL worker groups, workers establish reproductive dominance hierarchies via aggressive interactions. Aggression precedes ovarian activation [54,56] and thus aggressive interactions can be used to determine a bee’s position in the dominance hierarchy before ovarian activation is completed.

**Figure 1:**
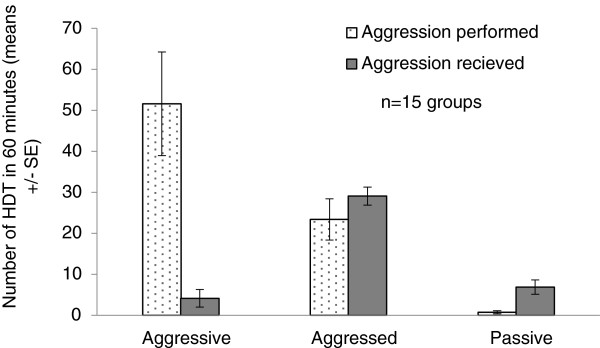
**Aggression in queenless worker groups.** The number of aggressive behaviors (sum of humming, darting and attack events) during 60 minutes performed or received by callow workers that were kept for 4 days. Observations were performed during days 3-4 after group establishment. Each group is composed of full sisters originating from a different colony (except for one, n = 15 groups, 14 colonies). Data are presented as means ± SE.

We established QL worker groups using callow (<24 hour old) workers (7 workers/cage). Previous behavioral observations revealed that workers could be classified on the basis of aggressive interactions during days 3 and 4 after group establishment [54]. We therefore collected these groups for analyses at the end of day 4. We identified three categories of workers in each cage: the most aggressive (1st in hierarchy), the most aggressed (2nd in hierarchy), or the most passive bee (7th in hierarchy). Passive workers performed aggressive behaviors least frequently. Among the two top bees in the hierarchy, the aggression exhibited by the aggressive (1st) bee was almost twice as frequent as that exhibited by the aggressed (2nd) bee. The aggressed (2nd) workers received a higher amount of aggression compared to the other two bee categories (Figure 1). Judging from the low aggression that the aggressive (1st) bee received, we can conclude that the majority of the aggression exhibited by the aggressed (2nd) bee was towards the other bees in the group and not towards the most aggressive (1st) bee in the hierarchy.

Since the workers were only 4 days old when sacrificed they all had relatively low ovarian activation, with oocytes averaging less than 0.5 mm compared to the size of ~3 mm of ready-to-lay eggs in *B. terrestris*. There were no significant differences between the mean oocyte sizes of the three bee categories (0.49 ± 0.04, 0.42 ± 0.04, and 0.31 ± 0.04 mm for 1st, 2nd and 7th bees, respectively (14 groups); Kruskal-Wallis test H (2, n = 42) = 5.97, p > 0.05).

**Figure 2:**
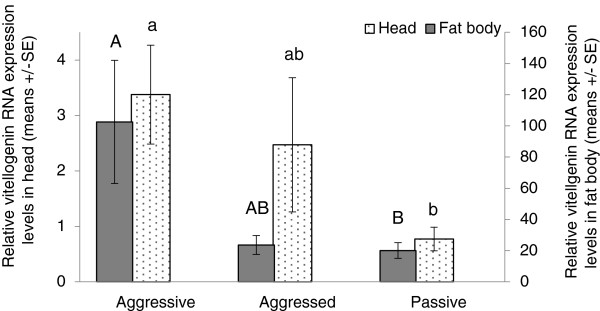
**The effect of aggression on the relative *vg *RNA expression levels in queenless groups.** Callow workers were kept in seven worker groups for 4 days. The “aggressive” individual was the most dominant worker that performed the highest number of aggressive behaviors during days 3-4; the “aggressed” individual was the worker that received the highest number of aggressive behaviors; and the “passive” worker performed and received the lowest number of aggressive behaviors per group. Each group was composed of full sisters originating from a different colony (except for one, n = 15 groups, 14 colonies). The relative amounts of *vg* in the heads were tested in all 15 groups and in the fat body in 5 groups. Data are presented as means ± SE. Different letters above the columns represent statistical differences at α = 0.05.

Significant differences were found in the *vg* RNA expression levels between aggressive (1st) and passive (7th) worker categories both in the head and in the abdominal fat body, with the aggressive bees possessing the highest *vg* expression levels (head: Kruskal-Wallis test: H(2, n = 45) = 7.12, p = 0.02 followed by multiple comparison post hoc test p = 0.02 for passive vs. aggressive. Abdominal fat body: Kruskal-Wallis test: H(2, n = 14) = 6.76, p = 0.03 followed by multiple comparison post-hoc test p = 0.03 for aggressive vs. passive). The aggressed (2nd) workers had intermediate levels of *vg* expression that were not statistically significantly different from either the aggressive (1st) or the passive (7th) worker categories (Figure 2).

### Experiment 4: Examining the effect of juvenile hormone and social interactions (aggression) on *vg* RNA expression levels in workers

Here, we compared aggression, ovarian activation, and *vg* expression levels in JH-treated, Dimethylformadide (DMF) treated (solvent control), and untreated control workers (“treatment”) that were introduced into either peer or older established groups (“group-type”). We hypothesized that workers introduced into peer groups (i.e., workers of the same age as the introduced worker) would have an equal probability of becoming dominant as any of the other workers; whereas workers introduced into established groups (i.e., of workers that are four days older than the introduced worker) are more likely to remain subordinate. Thus, workers introduced into peer groups are expected to receive and perform more aggressive behaviors while attempting to achieve dominance, and to activate their ovaries more rapidly compared to workers introduced into the established groups. We also hypothesized that JH treatment should stimulate *vg* expression and aggression (and thus ovarian activation) in both contexts, but more profoundly in the peer groups in which the social context is more tolerant to dominant behaviors.

Two experiments were performed. In the first we examined the effect of group type on ovarian activation by introducing callow workers into the two group types and examining their ovarian activation 7 days post-introduction. Workers that were introduced into established groups had lower ovarian activation relative to that of workers that were introduced into peer groups (T-test: t = 2.46, n_1_ = 15, _n2=_16, p = 0.01) (Table 3). This indicates that our manipulation of social state (peer/established) impacted the reproduction of the introduced workers, and thus their ability to achieve reproductive dominance. In the second experiment we examined the effects of treatment (JH/DMF/untreated control) and group type (peer/established) on ovarian activation, *vg* RNA expression levels, and aggressive interactions over a four-day post-introduction period.

**Table 3:** Behavior and ovarian activation in workers introduced to peer and experienced groups

	**Peer groups**			**Experienced groups**			**P value**
	**Control**	**DMF**	**JH**	**Control**	**DMF**	**JH**	
Total HDT per group	70.8±10	48.9±15.1	52.1±16.8	50.75±12.2	54.2±12.1	37.3±7.3	p=0.56; p=0.34^1^
	n=10	n=10	n=7	n=8	n=10	n=9	
HDT performed by IW	36.9±13.6	30.6±12.1	35.7±15	14.2±3.5	6.3±2.3	4.5±1.1	p=0.97; **p=0.02**^**2**^
	n=10	n=10	n=7	n=8	n=10	n=9	
HDT received by IW	32.4±8.2	33.7±7.1	20.3±6.8	40.5±3.5	42.1±6.9	42±5.2	p=0.61; p=0.29^3^
	n=10	n=10	n=7	n=8	n=10	n=9	
Percentage of HDT in which IW was involved	50.6±10.9%	35.8±15.8%	32.1±13.4%	26.25±6.1%	26.9±7.4%	17.4±3.9%	P=0.84; **p=0.026**^**4**^
	n=10	n=10	n=7	n=8	n=10	n=9	
Ovarian activation of IW (4 days)	0.14±0.01	0.12±0.01	0.17±0.01	0.17±0.02	0.16±0.02	0.13±0.02	p=0.13; p=0.68^5^
	n=29	n=28	n=26	n=8	n=10	n=9	
Ovarian activation of IW (7 days)	2.24±0.2	-	-	1.47±0.23	-	-	t-test: t=2.46, **p=0.01**
	(n=15)			(n=16)			

The introduced worker was assigned to one of three treatments: (1) 100ug JH-III diluted in 5 ul DMF applied to the thorax before introduction (2) 5ul DMF (3) untreated control. All workers were callow when introduced. Groups were composed of full sisters originated from the same colony (20 colonies in total) Statistical comparison was done using a nested ANOVA test. ‘IW’ refers to the introduced worker. HDT refers to the sum of humming, darting and attack behaviors received or performed by the introduced worker

^1^Nested ANOVA, treatment is nested in group-type: f(4,48) = 0.7, p = 0.56, For group-type only: f(1,48) = 0.91, p = 0.34.

^2^Nested ANOVA, treatment is nested in group-type: f(4,48) = 0.12, p = 0.97; For group-type only: f(1,48) = 5.4, p = 0.02.

^3^Nested ANOVA, treatment is nested in group-type: f(4,48) = 0.67, p = 0.61; For group-type only: f(1,48) = 1.14, p = 0.29.

^4^Nested ANOVA, treatment is nested in group-type: f(4,48) = 0.35, p = 0.84; For group-type only: f(1,48) = 5.27, p = 0.026. Data was transformed using arcsin.

^5^Nested ANOVA, treatment is nested in group-type: f(4,77) = 1.83, p = 0.13; For group-type only: f(1,77) = 0.16, p = 0.68.

Since ovarian activation in callow workers takes about 7 days [57,58] it was accordingly low in the examined 4-day-old workers and not significantly different between group type or treatments for all group-types and all treatments (Nested ANOVA, treatment is nested in group-type: f(4,77) = 1.83, p = 0.13; for group-type only: f(1,77) = 0.16, p = 0.68).

**Figure 3:**
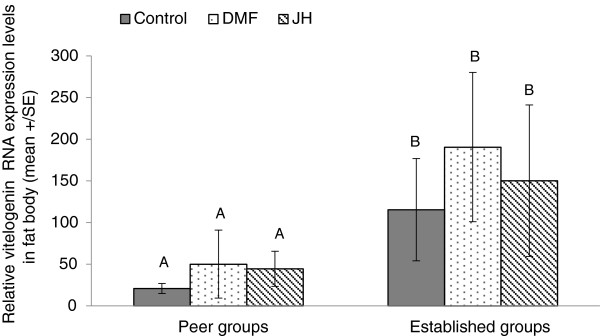
**Relative *vg *RNA expression levels under JH treatment in different social contexts.** Four-day-old workers were introduced into either peer or established groups (9 and 5 workers in each category in peer and established groups, respectively) and were assigned to one of three treatments: (1) 100 ug JH-III diluted in 5 ul DMF applied to the thorax before introduction; (2) 5 μl DMF; and (3) untreated control groups. All workers were callow when introduced and were kept for 4 days. Groups were composed of full sisters originating from the same colony. Each group was taken from a different colony. Data are presented as means ± SE. Different letters above the columns represent statistical differences at α = 0.05.

Within a group type there was no effect of treatment (JH, DMF or untreated control) on *vg* expression levels in the abdominal fat body (Figure 3) or head (data not shown). Interestingly, however, there were significant differences between the groups types. Levels of *vg* in workers introduced into the established groups were higher than in those introduced into the peer groups, irrespective of treatment (Nested ANOVA, treatment is nested in group-type: f(4,36) = 0.29, p = 0.87; for group-type only: f(1,36) = 8.06, p = 0.007) (Figure 3).

Treatment (JH, DMF and untreated) did not affect the level of aggression performed or received by the introduced worker in either peer or established groups (see statistics in Table 3). However, there were differences in aggression as a function of group-type. The total aggression (*i.e.* the sum aggression that was performed by all three workers in each group) was not significantly different among groups, irrespective of the group-type or treatment (nested ANOVA, treatment is nested in group-type: f(4,48) = 0.7, p = 0.56; for group-type only: f(1,48) = 0.91, p = 0.34). Nonetheless, workers that were introduced into peer groups were significantly more aggressive towards their group-mates and were involved in more aggressive behaviors compared to workers that were introduced into the older established groups (Aggression performed: Nested ANOVA, treatment in nested in group-type: f(4,48) = 0.12, p = 0.97; for group-type only: f(1,48) = 5.4, p = 0.02; Aggression involved: Nested ANOVA, treatment in nested in group-type: f(4,48) = 0.35, p = 0.84; for group-type only: f(1,48) = 5.27, p = 0.026). However, the amount of aggression that the introduced workers received was comparable, irrespective of the group type into which they were introduced to (Nested ANOVA, treatment in nested in group-type: f(4,48) = 0.67, p = 0.61; for group-type only: f(1,48) = 1.14, p = 0.29).

## Discussion

It has been hypothesized that complex social behaviors evolved by adapting and modulating existing genetic “toolkits” so that they are expressed in novel contexts [1]. In the case of toolkits involving key genes in endocrine signaling pathways, changes in expression may produce profound changes in morphology, physiology, or function. Two outstanding examples of this model are vitellogenin and juvenile hormone in adult insects. These two factors play critical roles in shaping reproductive processes in many solitary insects [24-26], but have been co-opted to function in maturation and division of labor in *Apis mellifera* honey bee workers [10,40]. Furthermore, the molecular interactions between these two factors have undergone a fundamental alteration in the honey bee: in the ancestral state, JH appears to positively regulate vitellogenin transcription (*vg)*, but in the honey bee JH and *vg* appear to function in a negative feedback loop [36,38-40]. Here, we examine the function and interactions of JH and *vg* in a primitively eusocial species, *B. terrestris*, in which JH has retained its ancestral gonadotropin role. We demonstrate that JH and *vg* expression are uncoupled, and that *vg* expression appears to be more tightly associated with social context than with reproductive physiology in workers. These results suggest that *B. terrestris* represents an intermediate stage in the evolution of the JH-vg pathway, from its ancestral role in reproduction to its new role in mediating worker social interaction and division of labor in advanced eusocial species.

### *Vg* role in *B. terrestris*

Our studies suggest that *vg* expression levels in *B. terrestris* are primarily associated with caste and social context, and are not strongly correlated with ovarian activation in workers. Expression levels of *vg* were higher in active queens than in workers, which is consistent with studies in honey bees [38], and with the superiority of the *B. terrestris* queen over workers in terms of egg production. Unlike the honey bee, however, *vg* expression levels were not associated with worker task. Finally, while *vg* expression levels were associated with reproductive state in queens, their levels were not strongly associated with reproductive state in workers. When correlations between *vg* and ovarian activation were observed in workers, they were likely to be mediated through aggressive behavior. Since aggression precedes ovarian activation in bumble bees [54,56], these two factors are closely intertwined. Selecting younger bees that differ in aggression levels but have inactive ovaries allowed us to disentangle the effects of aggression and ovarian activation, and demonstrated that expression of *vg* is higher in aggressive workers even before ovarian activation takes place. Thus, *vg* expression primarily correlates with aggression and only secondarily with reproduction. This is consistent with studies in the honey bee in which *vg* expression levels do not differ between sterile and reproductive workers [59], or between virgin queens and instrumentally inseminated queens with activated ovaries [60].

These inconsistent correlations of *vg* with reproduction can be explained in several ways: first, vitellogenin may evolve to function beyond the restricted context of reproduction [10,61]. Recent studies in honey bees showed that vitellogenin has multiple coordinating effects on social organization, such as pacing the onset of foraging behavior, priming bees for specialized foraging tasks, and influencing worker longevity [62]. Vitellogenin in honey bees was further suggested to reflect the nutritional status of the bee [63], and is associated with changes in gustatory responsiveness [64] and metabolism [65]. In several other species, vitellogenin can reduce oxidative stress by scavenging free radicals and promote longevity [38,66]. Recently, vitellogenin was linked with immune defense and was suggested to be produced under immune challenge. In fish, vitellogenin has been shown to recognize pathogens and to promote macrophage phagocytosis [61,67]; and in the honey bee, vitellogenin is downregulated in parasite infested bees [68]. These studies suggest that vitellogenin has many divergent functions in different species, and is not restricted solely to reproduction.

A second explanation for the inconsistencies in correlations is that vitellogenin may have evolved to regulate reproduction indirectly. In solitary species where there is no competition over reproduction, JH may directly regulate *vg* levels, which in turn directly regulate ovarian activation. However, in primitively social species, social groups are composed of both dominant and subordinate females that compete over reproduction, and egg-laying is monopolized by the most aggressive females. Thus, a double control system, in which *vg* responds to aggression levels instead of or in addition to JH levels, may be useful in order to prevent the subordinate females from overloading their system with higher *vg* levels when reproduction is not feasible. A recent study in the harvester ant, *Pogonomyrmex barbatus harbors,* may support this hypothesis: in this species, the gene for vitellogenin has been duplicated and subfunctionalized to acquire reproductive and non-reproductive functions - mainly behavioral - in sterile workers [69].

A third possible explanation is that vitellogenin’s mode of action masks the positive correlation between *vg* levels and reproduction. For example, regulation may occur at the level of translation to the protein, or at the rate at which the circulating protein is sequestered in the developing oocytes. For example, in grasshoppers preparing for oviposition, high levels of *vg* mRNA persist but translation diminishes [24,27,30]. A similar mechanism was suggested for *Apis mellifera*[70]. If a similar mechanism operates in *B. terrestris*, *vg* mRNA levels would remain high while protein levels decrease in workers with almost fully developed ovaries. This may also explain why 5- and 8-day-old workers do not differ in their *vg* levels despite large differences in their ovarian activation. Further studies are needed to determine whether these hypothesized alternative regulatory mechanisms are operating.

### Interaction between JH and *vg* in *B. terrestris*

We found no effect of increasing JH titers on *vg* expression in worker bees. Since JH-vg interactions may be dynamic, it is possible that the timepoint we used (4 days post JH treatment) did not fully capture any effects on *vg*. However, previous study at this timepoint demonstrated that JH treatment resulted in significant changes in brain expression levels in *B. terrestris*[71]. Furthermore, we selected this timepoint because it takes seven days for workers to produce fully mature ooctyes, and thus the oocytes of four day old workers are immature. This is critical for two reasons: first, the correlation between *vg* mRNA and vitellogenin titer occurs only during the oocyte growth period, rather than when the oocyte has reached its ready-to-lay size [24,27,30]. Second, JH does not induce an immediate response at the vitellogenin protein levels, but a progressive one. In *Locusta migratoriaIn,* for example, vitellogenin shows only a small increase in the first 48 hours post-treatment and then steep increases to its maximum at 72 hours. Thereafter it gradually declines [30,35]. However, though we selected this time point to optimally capture any effects of JH on *vg*, it is possible that the effects occurred at an earlier or later time point. Furthermore, since we did not measure JH levels in the hemolymph of the bees, we cannot preclude the possibility that the lack of effect of JH was due to its post-application degradation. Finally JH may exert its primary effects on other endocrine glands or tissues, and only indirectly affect vitellogenin synthesis [24], which should be taken into consideration when measuring *vg* RNA levels in response to JH. Nonetheless, despite choosing the seemingly optimal time point, JH application did not appear to have any effect on *vg* expression.

According to these findings, either both JH and *vg* are regulated by a third party; or JH regulation of *vg*, if exists, must be indirect. The correlation of *vg* with aggression, the fact that aggression precedes ovarian development (the aggressive worker is the most fertile one in more than 90% of groups [55]), and that JH affects ovarian activation in workers (but not aggression) [47,72], lend credence to the hypothesis that both JH and *vg* are regulated by a third player. Alternatively, it is possible that the gonadotropin effect of JH in *B. terrestris* is separated from the association between *vg* and aggression, and that both pathways (*vg*-aggression and JH production) independently but jointly affect ovarian activation.

What might be the selective advantages of two disparate mechanisms for regulating reproduction? The queen presumably inhibits worker reproduction through reducing their JH levels [47]. Even when worker reproduction occurs, only about 40% of the workers lay eggs, although about 70% have activated ovaries [73]. Since resources are limited and not all the eggs can develop to adults, egg-laying workers gain their position through aggressive interactions and establishment of a dominance hierarchy [54,56]. Thus, access to reproduction requires detection of both the right social condition of the colony (cooperative vs. competition) and the right social context among workers (dominant vs. subordinate). While the first can be achieved through manipulating the JH levels in subordinates by the queen or dominant workers, the second can be mediated through aggressive interactions that affect *vg* levels and thus determine which bees will reproduce. Such a double control ensures that efforts will be invested in reproduction only when feasible.

### Effect of social context on JH-*vg* interactions

While *vg* levels were not affected by JH, they were affected by social context. Interestingly, *vg* levels were higher in bees introduced into established groups although they exhibited less aggression compared to those introduced into peer groups. This is in contrast to the finding that *vg* levels were higher in aggressive compared to passive workers (Figures 1 and 2). One explanation is that it is not only the aggression performed by workers that matters, but also the aggression they receive. Workers that were introduced into the established groups in experiment 4 had higher *vg*, performed significantly less aggression, and received slightly more aggression (although not statistically significant Tables 1 and 2, Figure 3), while both aggressive and aggressed workers in experiment 3 expressed higher levels of *vg* (Figures 1 and 2). Thus, it is possible that *vg* is higher in both the performer and receiver of aggression (but not in passive workers). This hypothesis is supported by ample evidence from the bees’ life cycle. For example, larger groups of workers, in which aggression is higher, are more productive (in terms of ovarian activation and egg laying) compared to smaller ones [55]. Recently published studies conducted with paired bees of *B. impatiens* have suggested that aggression in one worker stimulates aggression in the other [74], though whether *vg* levels rise in both workers remains to be determined. Finally, newly mated queens are stimulated to lay eggs in the presence of other queens or workers, with egg-laying following a period of intensive aggressive interactions (personal observations, EA). However, this hypothesis still remains to be tested directly.

## Conclusions

Here, we comprehensively examined the interactions between JH and vitellogenin in a primitively eusocial species, *B. terrestris*. We have demonstrated that *vg* expression levels are higher in queens than in workers, and in laying versus virgin queens, but that levels in workers are not associated (or only partially associated) with age, ovarian activation, task, or queen presence. Interestingly, however, there is a strong association between expression levels of *vg* in workers and the amount of aggression performed or received. Furthermore, JH does not regulate *vg* levels in queenless workers. Our results suggest that although JH has retained its traditional role as a gonadotropin in bumble bees, vitellogenin has already been co-opted into a novel role and is regulated by different, uncharacterized, physiological factors, which, in turn, may be regulated by aggression. Further studies in bumble bees may reveal the molecular and physiological mechanisms by which the *vg*-JH pathway became uncoupled in this system.

## Methods

### Bees

Colonies of *B. terrestris* (Yad Mordechai Apiary, Israel) were obtained 3–5 days after the first worker had emerged. They were maintained in the laboratory in nest boxes (23 × 23 × 10 cm) at a constant temperature of 30°C and 50%–60% relative humidity, and supplied *ad libitum* with a sugar solution and fresh pollen collected from honey bee colonies. In all the experiments described below, all workers from within a group were taken from the same colony and different groups were taken from different colonies (in total we used 40 different colonies in the current study). This was done in order to reduce the variability within groups while maximizing biological variation among replicates. All the bees were sacrificed by freezing on dry ice and stored at -80°C. We examined *vg* levels (either head or fat body or both) and ovarian activation in all bees.

### Assessment of ovarian activation levels

Individual bees were dissected under a stereo-microscope in double-distilled water. The length of the terminal oocyte in the three largest ovarioles (at least 1 ovariole per ovary; workers possess 4 ovarioles per ovary) was measured with a scaled ocular. Mean terminal oocyte length for each bee was used as an index of ovarian activation [54-56,75].

### Identification of vitellogenin gene in *B. terrestris*

We identified the bumble bee *vg* gene using the NCBI/blast home page (http://blast.ncbi.nlm.nih.gov/Blast.cgi). The gene for vitellogenin was annotated and characterized in *B. ignitus*[76] and *B. hypocrite*[77], two species very closely related to *B. terrestris*. We searched for homologs to the *B. hypocrita vitellogenin* gene in the *B. terrestris* database (GenBank accession number taxid: 30195). The deduced *vitellogenin* sequence of *B. terrestris* (NCBI Reference Sequence: XR_131915.1) was aligned with that from *B. hypocrita* (taxid: 130701) with 99% query coverage and E-value of <0.0001 and with that of *B. ignitus* (taxid: 130704) with 99% query coverage and E-value of <0.0001.

Sequences of *vitellogenin* orthologs were obtained for three *Bombus* species and *Apis mellifera* (Additional file 1, GenBank accession numbers for all orthologs can be found below). The complete protein sequences (translated from mRNA) were aligned using ClustalW2 packaging in BioEdit 7.2.0, using the full multiple alignment feature. Alignment parameters were set to Bootstrap NJ tree with number of bootstrap set to 1000. Open reading frame for *B. terrestris* was selected between nucleotide 11 to 5350 using ORF finder. Accession numbers were as follow: *Bombus terrestris*: XR_131915.1; *Bombus hypocrite*: GQ340749.1; *Bombus ignitus*: FJ913883.1; *Apis mellifera:* NM_001011578.1.

### Quantification of *vg* expression levels

Total RNA was extracted from the head or abdominal fat body of individual bumble bees, either queens or workers, using the RNeasy mini kit (Qiagen, Valencia, CA) according to the manufacturer’s instructions. Abdominal fat body contained the abdomen cuticle and the fat body attached it, while the contents of the abdomen, including intestine, ovaries, and sting complex, were removed during dissection on dry ice. RNA quantity and quality were assayed with a ND-1000 Spectrophotometer (NanoDrop Technologies, Wilmington DE). cDNA was synthesized according to the manufacturer’s instructions using 500 ng of RNA with Reverse Transcriptase (Invitrogen, Carisbad, CA, USA). The first strand cDNA reaction was diluted by adding 35 μl of ultra-purified water and stored at -20°C until use.

Two microliters of diluted cDNA were used together with 5 μl SYBR-Green (Bioline, Luckenwalde, Germany), 0.2 mM of each gene specific primer and 2.6 μl DEPC-water for the gene expression assay. Primer design for *vg* (Fwd: 5′AAGAATCATCTGAGCAACGTGA 3′; Rev: 5′TAGTGCACTGTTTGCTTTTGGT 3′) was performed using the Primer3 v 0.4.0 (http://frodo.wi.mit.edu/). To control for PCR efficiency and individual differences across samples, two housekeeping genes were used: Arginine kinase (*AK*) (Fwd: 5′ TGTCGGTATCTACGCGCCTG 3′; rev: 5′ TTGGTGGATGCTTGTCAGTC 3′) and Phospholipase A2 (*PLA2*) (Fwd: 5′ GGTCACACCGAAACCAGATT 3′; rev: 5′ TCGCAACACTTCGTCATTTC 3′) [78]. Expression levels of *vg* were determined using quantitative real-time PCR on an ABI Prism®7900 sequence detector with SYBR Green detection method (Applied Biosystems, Foster City, CA, USA). Triplicate reactions were performed for each of the samples and averaged for use in statistical analysis. Quantification was based on the number of PCR cycles required to cross a threshold of fluorescence intensity (Ct), using the 2^-∆Ct^ technique. The geometric mean of the two reference genes was used as a control. Negative control samples (cDNA reaction without RT enzyme) and a water control were also present on each plate. PCR product quality and specificity was verified using melt curve analysis. A standard curve was performed for each set of primers using 5 different concentrations of cDNA in order to determine the r (>0.96 for all primers) and efficiency (98%, 83%8 and 86% for AK, PLA2 and vg, respectively).

### Experiment 1: Examining the association of vg expression with caste, ovarian activation and worker task in *B. terrestris*

We examined *vg* levels (heads) and ovarian activation in fertile and sterile workers and queens. Sterile QR workers (n = 6) were sampled from 6 different full-sized colonies. In each colony we individually marked callow workers (< 24 h old), returned them to their original colony and collected them 4 days later. From the same colonies we removed callow sister-workers and kept them in QL groups (2 or 3 bees/group) for 10 days (n = 6 groups). We collected 6 virgin queens (3-days-old or older) and 6 mated queens from the same colonies. To reduce the variability between subordinates and dominant bees in the QL groups, we always selected the reproductively dominant bee (based on her ovarian activation compared to her nestmates) for any analysis we performed.

To examine the association of worker task with *vg* expression, we determined task, *vg* levels (head) and ovarian activation in foragers and nurses that were sampled from two additional colonies. These colonies were first kept under lab rearing conditions (i.e., not allowed to forage freely), and all emerging workers were tagged daily. Twenty days after first worker emergence, when the colonies contained at least 30 workers, food was removed progressively and the colonies were allowed to forage freely outside. The bees were allowed to acclimate to the free foraging conditions for one day, after which we monitored both foraging and nursing activities for 4 days. Foraging events were documented by observing the nest-entrance twice a day (6:00-7:00 am and 6:30-7:30 pm) and the in-nest activities were observed for 1 hour each day. The numbers of foraging and nursing events during the entire period of observations were summed for each worker individually (for more details see [75]). Four pairs of bees of the same age, one forager and one nurse in each pair, from each colony (8 pairs in total) were sampled.

### Experiment 2: Uncoupling the effects of age, ovarian activation and queen presence/absence on *vg* levels in workers

We examined *vg* levels (head) and ovarian activation in QR and QL workers that were sampled from 4 different full-sized colonies. In each colony we individually marked callow workers, returned them to their original colony and collected them 5 or 8 days later (QR group). From the same colonies we removed callow sister workers and kept them in QL groups (2 or 3 bees/group) for 5 or 8 days (n = 4 groups). From each group we selected the reproductively dominant bee based on assessment of ovarian activation as described above.

### Experiment 3: Examining the impact of social interactions (aggression) on *vg* levels in queenless worker groups

For this experiment, we measured *vg* levels (head and fat body), ovarian activation and aggression in 15 groups of 7 workers, each taken from 14 full-sized colonies. The groups were established using callow sister workers of approximately the same size from each colony and placing them in small nest boxes. Groups were observed for 10 minutes each, 3 times a day (morning: 9:00-11:00 am; noon: 12:00-2:00 pm; evening: 5:00-7:00 pm) during days 3 and 4 (a total of 60 minutes per group) and three antagonistic behaviors were monitored: humming, darting, and attack (for definitions see [54]). On the fourth day we selected, based on the sum of aggressive behaviors that each individual worker performed and received, the most aggressive (1st in hierarchy), the most aggressed (2nd) and the least aggressive (7th) workers in each group. Expression levels of *vg* were measured in the heads (15 groups) and abdominal fat bodies (5 groups). Ovarian activation was measured in 14 groups only, due to a technical problem with one of the groups.

### Experiment 4: Examining the effect of juvenile hormone and social interactions (aggression) on *vg* levels in workers

Here, we measured *vg* levels (head and fat body), ovarian activation and aggression in order to test the effects of JH treatment on expression levels of *vg* under two different social contexts, “peer groups” and “established groups”. For the peer groups, we introduced each callow worker into a group composed of two sister callow workers of the same age and approximately of the same size. Since all three workers in the peer groups were callows, the introduced worker, if left untreated, had an equal probability to show aggression (and thus, to gain dominance) compared to her nest mates. If treated with JH prior to her introduction, on the other hand, it was hypothesized that the treated bee would show higher aggression and thus have greater probability of becoming dominant. For the established groups, a callow worker was introduced into a group composed of two equal-size but 4-days-older sister workers kept as QL pair. Thus, the callow worker was introduced into a situation in which the other two workers had already established an aggressive-aggressed relationship [54], and therefore the callow worker had a lower chance to show aggression and gain dominance. We hypothesized, however, that treatment with JH would upgrade her position in the hierarchy and consequently the likelihood that she would show aggression. The introduced workers in each of the group types were assigned randomly to one of the following treatments: untreated control, treated with 5ul of dimethylformamide (solvent control, DMF, J.T Backer cat: 7032), or treated with 100 μg JH dissolved in 5 μl DMF (JH-III, Sigma, cat J2000, purity ≥ 65%). JH or DMF were topically applied to the dorsal part of the thorax, as in [71] but see also [47,79]. This treatment methodology using a slightly lower dose (70 μg) was sufficient to alter gene expression at a 4 day time point [71]. During days 3 and 4 post-introduction we monitored the aggressive behavior that was performed and received by the introduced worker and the total aggression exhibited in each group. The introduced bees were sampled after 4 days.

For this study, we used in total 14 colonies from which we created 110 groups (83 peer groups divided into 28 control, 29 DMF, 26 JH; 27 experienced groups divided into 8 control, 10 DMF, 9 JH). We analyzed only the introduced bees in each group. We collected data on ovarian activation in 83 introduced workers (65 peer and 15 experienced groups), monitored the behavior in 56 groups (27 peer and 27 experienced groups), and quantified *vg* in the heads of 15 introduced workers (only peer groups), and in the abdominal fat bodies of 68 workers (53 peer and 15 experienced groups). As additional control, we used another 3 colonies from which we created only untreated groups, either peer (n = 15 groups) or experienced (n = 16 groups), and kept the introduced workers for 7 days in order to test for any long term differences in ovarian activation of the workers that were introduced into these two groups.

### Statistical analysis

The data were tested for normality using a Kolmogorov-Smirnov test for normality. We used non-parametric tests each time the data sets were not normally distributed or sample size was small (<8 individuals). To test the effect of caste, ovarian activation, age and social condition on *vg* expression levels in experiments 1 and 2, we used a Mann-Whitney test. Since foragers and nurses were sampled as matched pairs of the same age, we used a Wilcoxon paired t-test to compare the differences related to task. The effect of JH treatment in peer vs. experienced groups in experiment 4 was compared using a nested ANOVA (treatment (JH, DMF, untreated) nested in group type (peer/experienced). When data are presented as percentages/proportions, they were transformed using arcsin before performing the parametric tests (in the cases where parametric tests were used). Data are presented as mean ± SE. Significant differences were accepted at α = 0.05.

### Availability of supporting data

The data sets supporting the results of this article are included within the article and its Additional file 1.

## Competing interests

The authors declare that they have no competing interests.

## Authors’ contributions

EA carried out the behavioral, physiological, and molecular analyses, including the sequence and phylogenetic analyses, designed the study and wrote the paper. OM participated in designing the molecular analysis and dissections. CMG and AH designed the study and wrote the paper along with EA. All authors read and approved the final manuscript.

## Supplementary Material

Additional file 1 **Alignments of the insect vitellogenin orthologs.** Alignment of vitellogenin orthologs from 3 bumble bees species and *Apis mellifera.* Click here for file

## References

[B1] Toth AL Varala K Newman TC Miguez FE Hutchison SK Willoughby DA Simons JF Egholm M Hunt JH Hudson ME Robinson GE Wasp gene expression supports an evolutionary link between maternal behavior and eusociality Science 2007 318 5849 441 444 10.1126/science.1146647 17901299

[B2] Robinson GE Vargo EL Juvenile hormone in adult eusocial Hymenoptera: gonadotropin and behavioral pacemaker Arch Insect Biochem Physiol 1998 35 4 559 583 9210289 10.1002/(SICI)1520-6327(1997)35:4<559::AID-ARCH13>3.0.CO;2-9

[B3] Nijhout HF Wheeler D Juvenile hormone and the physiological basis of insect polymorphism Q Rev Biol 1982 57 2 109 133 10.1086/412671

[B4] Hartfelder K Insect juvenile hormone: from “status quo” to high society Brazil J Med Biolog Res 2000 33 2 157 177 10.1590/S0100-879X2000000200003 10657056

[B5] Brent CS Schal C Vargo EL Endocrine changes in maturing primary queens of *Zootermopsis angusticollis* J Insect Physiol 2005 51 11 1200 1209 10.1016/j.jinsphys.2005.06.009 16081092

[B6] Elliott KL Stay B Juvenile hormone synthesis as related to egg development in neotenic reproductives of the termite *Reticulitermes flavipes*, with observations on urates in the fat body Gen Comparat Endocrinol 2007 152 1 102 110 10.1016/j.ygcen.2007.03.003 17434168

[B7] Elliott KL Stay B Changes in juvenile hormone synthesis in the termite *Reticulitermes flavipes* during development of soldiers and neotenic reproductives from groups of isolated workers J Insect Physiol 2008 54 2 492 500 10.1016/j.jinsphys.2007.11.008 18187146

[B8] Cornette R Gotoh H Koshikawa S Miura T Juvenile hormone titers and caste differentiation in the damp-wood termite *Hodotermopsis sjostedti* (Isoptera, Termopsidae) J Insect Physiol 2008 54 6 922 930 10.1016/j.jinsphys.2008.04.017 18541259

[B9] Korb J Hoffmann K Hartfelder K Endocrine signatures underlying plasticity in postembryonic development of a lower termite, *Cryptotermes secundus* (Kalotermitidae) Evol Dev 2009 11 3 269 277 10.1111/j.1525-142X.2009.00329.x 19469854

[B10] Guidugli KR Nascimento AM Amdam GV Barchuk AR Omholt S Simoes ZL Hartfelder K Vitellogenin regulates hormonal dynamics in the worker caste of a eusocial insect FEBS Lett 2005 579 22 4961 4965 10.1016/j.febslet.2005.07.085 16122739

[B11] Amdam GV Page RE The developmental genetics and physiology of honeybee societies Anim Behav 2010 79 5 973 980 10.1016/j.anbehav.2010.02.007 20514137 PMC2875690

[B12] Sommer K Holldobler B Rembol H Behavioral and physiological aspects of reproductive control in a Diacamma species from Malaysia (Formicidae, Ponerinae) Ethology 1993 94 162 170

[B13] Dong SZ Ye GY Guo JY Hu C Roles of ecdysteroid and juvenile hormone in vitellogenesis in an endoparasitic wasp, *Pteromalus puparum* (Hymenoptera: Pteromalidae) Gen Comparat Endocrinol 2009 160 1 102 108 10.1016/j.ygcen.2008.11.007 19032957

[B14] Delisle J Cusson M Juvenile hormone biosynthesis, oocyte growth and vitellogenin accumulation in *Choristoneura fumiferana* and *C. rosaceana*: a comparative study J Insect Physiol 1999 45 6 515 523 10.1016/S0022-1910(98)00155-3 12770336

[B15] Panaitof SC Scott MP Effect of juvenile hormone on vitellogenin gene expression in the fat body of burying beetles, *Nicrophorus orbicollis* Arch Insect Biochem Physiol 2006 63 2 82 91 10.1002/arch.20144 16983666

[B16] Wang ZW Davey KG The role of juvenile hormone in vitellogenin production in *Rhodnius prolixus* J Insect Physiol 1993 39 6 471 476 10.1016/0022-1910(93)90078-6

[B17] Martin D Piulachs MD Belles X Patterns of hemolymph vitellogenin and ovarian vitellin in the German cockroach, and the role of juvenile hormone Physiol Entomol 1995 20 1 59 65 10.1111/j.1365-3032.1995.tb00801.x

[B18] Glinka AV Kleiman AM Wyatt GR Roles of juvenile-hormone, a brain factor and adipokinetic hormone in regulation of vitellogenin biosynthesis in *Locusta-migratoria* Int J Biochem Mol Biol 1995 35 2 323 328 7663387

[B19] Klowden MJ Endocrine control of mosquito reproduction Arch Insect Biochem Physiol 1997 35 491 512 10.1002/(SICI)1520-6327(1997)35:4<491::AID-ARCH10>3.0.CO;2-5

[B20] Parthasarathy R Sun ZY Bai H Palli SR Juvenile hormone regulation of vitellogenin synthesis in the red flour beetle, *Tribolium castaneum* Insect Biochem Mol 2010 40 5 405 414 10.1016/j.ibmb.2010.03.006 PMC2875371 20381616

[B21] Barth RH Lester LJ Sroka P Kessler T Hearn R Juvenile hormone promotes dominance behavior and ovarian development in social wasps (Polistes annularis) Experientia 1974 31 6 691 692 1170088 10.1007/BF01944632

[B22] Bloch G Borst DW Huang ZY Robinson GE Cnaani J Juvenile hormone titers, juvenile hormone biosynthesis, ovarian development and social environment in B. terrestris J Insect Physiol 2000 46 47 57 10.1016/S0022-1910(99)00101-8 12770258

[B23] Giray T Giovanetti M West-Eberhard MJ Juvenile hormone, reproduction, and worker behavior in the neotropical social wasp *Polistes canadensis* Proc Natl Acad Sci USA 2005 102 9 3330 3335 10.1073/pnas.0409560102 15728373 PMC552932

[B24] Chen TT Hillen LJ Expression of the vitellogenin genes in insects Gamete Res 1983 7 179 196 10.1002/mrd.1120070210

[B25] Hagedorn HH Vitellogenin and vitellin in insects Ann Rev Entomol 1979 24 475 505 10.1146/annurev.en.24.010179.002355

[B26] Engelmann F Treherne JE, Berridge MJ, Wigglesworth VB Insect vitellogenin: identification, biosynthesis and role in Vitellogenesis Advanced in Insect Physiology, vol. 14 1979 New York: Academic Press INC 49 108

[B27] Borst DW Eskew MR Wagner SJ Shores K Hunter J Luker L Hatle JD Hecht LB Quantification of juvenile hormone III, vitellogenin, and vitellogenin-mRNA during the oviposition cycle of the lubber grasshopper Insect Biochem Mol Biol 2000 30 8 813 819 10876125 10.1016/s0965-1748(00)00053-9

[B28] Adams TS Filipi PA Yi SX Effect of age, diet, diapause and juvenile hormone on oogenesis and the of amount of vitellogenin and vitellin in the two spotted stink bug, *Perillus bioculatus* (Heteroptera :pentatomidae) J Insect Physiol 2002 48 4 477 486 10.1016/S0022-1910(02)00069-0 12770097

[B29] Sueren-Castillo S Abrisqueta M Maestro JL FoxO inhibits juvenile hormone biosynthesis and vitellogenin production in the German cockroach Insect Biochem Mol Biol 2012 42 7 491 498 10.1016/j.ibmb.2012.03.006 22487089

[B30] Chinzei Y White BN Wyatt GR Vitellogenin mRNA in locust fat body: identification, isolation, and quantitative changes induced by juvenile hormone Can J Biochem 1982 60 243 251 6177387 10.1139/o82-029

[B31] Comas D Piulachs MD Belles X Fast induction of vitellogenin gene expression by juvenile hormone III in the cockroach *Blattella germanica* (L.) (Dictyoptera, Blattellidae) Insect Biochem Mol 1999 29 9 821 827 10.1016/S0965-1748(99)00058-2 10510500

[B32] Comas D Piulachs MD Belles X Induction of vitellogenin gene transcription in vitro by juvenile hormone in *Blattella germanica* Mol Cell Endocrinol 2001 183 1–2 93 100 11604229 10.1016/s0303-7207(01)00589-5

[B33] Sheng Z Xu J Bai H Zhu F Palli SR Juvenile hormone regulates vitellogenin gene expression through insulin-like peptide signaling pathway in the red flour beetle, *Tribolium castaneum* J Biolog Chem 2011 286 49 41924 41936 10.1074/jbc.M111.269845 PMC3234905 22002054

[B34] Nijhout HF Insect Hormones 1994 Princeton: Princeton University Press

[B35] Chen TT Couble P DeLuca FL Wyatt GR Juvenile Hormone Control of Vitellogenesis in Locusta migratoriaIn 1976 New York: Plenum Press

[B36] Amdam GV Omholt SW The regulatory anatomy of honeybee lifespan J Theor Biol 2002 216 2 209 228 10.1006/jtbi.2002.2545 12079372

[B37] Pinto LZ Bitondi MMG Simoes ZLP Inhibition of vitellogenin synthesis in *Apis mellifera* workers by a juvenile hormone analogue, pyriproxyfen J Insect Physiol 2000 46 2 153 160 10.1016/S0022-1910(99)00111-0 12770247

[B38] Corona M Velarde RA Remolina S Moran-Lauter A Wang Y Hughes KA Robinson GE Vitellogenin, juvenile hormone, insulin signaling, and queen honey bee longevity Proc Natl Acad Sci USA 2007 104 17 7128 7133 10.1073/pnas.0701909104 17438290 PMC1852330

[B39] Wang Y Brent CS Fennern E Amdam GV Gustatory perception and fat body energy metabolism are jointly affected by vitellogenin and juvenile hormone in honey bees PLoS Genet 2012 8 6 e1002779 10.1371/journal.pgen.1002779 22761585 PMC3386229

[B40] Amdam GV Norberg K Hagen A Omholt SW Social exploitation of vitellogenin Proc Natl Acad Sci USA 2003 100 4 1799 1802 10.1073/pnas.0333979100 12566563 PMC149913

[B41] Formesyn EM Cardoen D Ernst UR Danneels EL Vaerenbergh MV Koker DD Verleyen P Wenseleers T Schoofs L Graaf DCD Reproduction of honeybee workers is regulated by epidermal growth factor receptor signaling Gen Comparat Endocrinol 2014 197 1 1 4 10.1016/j.ygcen.2013.12.001 24333651

[B42] Pontona F Wilsonc K Holmesb AJ Cottere SC Raubenheimerf D Simpsona SJ Integrating nutrition and immunology: A new frontier J Insect Physiol 2013 59 2 130 137 10.1016/j.jinsphys.2012.10.011 23159523

[B43] Penick CA Liebig J Brent CS Reproduction, dominance, and caste: endocrine profiles of queens and workers of the ant *Harpegnathos saltator* J Comparat Physiol 2011 197 11 1063 1071 10.1007/s00359-011-0667-0 21773739

[B44] Cuvillier-Hot V Lenoir A Peeters C Reproductive monopoly enforced by sterile police workers in a queenless ant Behav Ecol 2004 15 970 975 10.1093/beheco/arh072

[B45] Maekawa K Ishitani K Gotoh H Cornette R Miura T Juvenile Hormone titre and vitellogenin gene expression related to ovarian development in primary reproductives compared with nymphs and nymphoid reproductives of the termiteReticulitermes speratus Physiol Entomol 2010 35 1 52 58 10.1111/j.1365-3032.2009.00711.x

[B46] Duchateau MJ Velthuis HHW Development and reproductive strategies in *Bombus terrestris* colonies Behavior 1988 107 186 207 10.1163/156853988X00340

[B47] Roseler PF Juvenile hormone control of oogenesis in bumblebee workers, *Bombus terrestris* J Insect Physiol 1977 23 985 992 10.1016/0022-1910(77)90126-3

[B48] Roseler PF Roseler I Influence of juvenile hormone on fat body metabolism in ovariolectomized queens of the bumblebee, *Bombus terrestris* Insect Biochem Mol Biol 1988 18 6 557 563 10.1016/0020-1790(88)90007-8

[B49] Van Doorn A Factors influencing dominance behavior in queenless bumblebee workers *Bombus terrestris* Physiol Entomol 1989 14 211 222 10.1111/j.1365-3032.1989.tb00954.x

[B50] Michener CD The Social Behavior of the Bees 1974 Cambridge, Massachusetts: Harvard University Press

[B51] Bourke AFG Worker matricide in social bee and wasps J Theor Biol 1994 167 283 292 10.1006/jtbi.1994.1070

[B52] Van Honk CJK Roeseler PF Velthuis HHW Hogeveen JC Factors influencing egg laying of workers in a captive *Bombus terrestris* colony Behav Ecol Sociobiol 1981 9 9 14 10.1007/BF00299847

[B53] Van Doorn A Heringa J The ontogeny of a dominance hierarchy in colonies of the bumblebee *Bombus terrestris* (Hymenoptera, Apidae) Insect Soc 1986 33 3 25 10.1007/BF02224031

[B54] Amsalem E Hefetz A The appeasement effect of sterility signaling in dominance contests among *Bombus terrestris* workers Behav Ecol Sociobiol 2010 64 10 1685 1694 10.1007/s00265-010-0982-4

[B55] Amsalem E Hefetz A The effect of group size on the interplay between dominance and reproduction in *Bombus terrestris* PloS One 2011 6 3 e18238 10.1371/journal.pone.0018238 21464893 PMC3065479

[B56] Amsalem E Shamia D Hefetz A Aggression or ovarian development as determinants of reproductive dominance in *Bombus terrestris*: interpretation using a simulation model Insect Soc 2013 60 2 213 222 10.1007/s00040-013-0285-7

[B57] Duchateau MJ Velthuis HHW Ovarian development and egg-laying in workers of Bombus-terrestris Entomologia Experimentalis Et Applicata 1989 51 3 199 213 10.1111/j.1570-7458.1989.tb01231.x

[B58] Amsalem E Twele R Francke W Hefetz A Reproductive competition in the bumble-bee *Bombus terrestris*: do workers advertise sterility? Proc Biol Sci 2009 276 1660 1295 1304 10.1098/rspb.2008.1688 19129137 PMC2660966

[B59] Grozinger CM Yongliang F Hoover SER Winston RL Genome-wide analysis reveals differences in brain gene expression patterns associated with caste and reproductive status in honey bees (*Apis mellifera*) Mol Ecol 2007 16 22 4837 4848 10.1111/j.1365-294X.2007.03545.x 17927707

[B60] Niño EL Tarpy DR Grozinger CM Differential effects of insemination volume and substance on reproductive changes in honey bee queens (*Apis mellifera L*.) Insect Mol Biol 2013 22 3 233 244 10.1111/imb.12016 23414204

[B61] Li Z Zhang S Vitellogenin functions as a multivalent pattern recognition receptor with an opsonic activity PloS One 2008 3 4 e1940 10.1371/journal.pone.0001940 18398466 PMC2277463

[B62] Nelson CM Ihle KE Fondrk MK Page RE Amdam GV The gene vitellogenin has multiple coordinating effects on social organization Plos Biol 2007 5 3 e62 10.1371/journal.pbio.0050062 17341131 PMC1808115

[B63] Amdam GV Omholt SW The hive bee to forager transition in honeybee colonies: the double repressor hypothesis J Theor Biol 2003 223 4 451 464 10.1016/S0022-5193(03)00121-8 12875823

[B64] Amdam GV Norberg K Page RE Jr Erber J Scheiner R Downregulation of vitellogenin gene activity increases the gustatory responsiveness of honey bee workers (*Apis mellifera*) Behav Brain Res 2006 169 2 201 205 10.1016/j.bbr.2006.01.006 16466813 PMC2398700

[B65] Wheeler MM Ament SA Rodriguez-Zas SL Robinson GE Brain gene expression changes elicited by peripheral vitellogenin knockdown in the honey bee Insect Mol Biol 2013 22 5 562 573 10.1111/imb.12043 23889463

[B66] Nakamura A Yasuda K Adachi H Sakurai Y Ishii N Vitellogenin-6 is a major carbonylated protein in aged nematode, *Caenorhabditis elegans* Biochem Biophys Res Commun 1999 264 580 583 10.1006/bbrc.1999.1549 10529405

[B67] Shi XD Zhang SC Pang QX Vitellogenin is a novel player in defense reactions Fish Shellfish Immunol 2006 20 769 772 10.1016/j.fsi.2005.09.005 16293421

[B68] Amdam GV Hartfelder K Norberg K Hagen A Omholt SW Altered physiology in worker honey bees (Hymenoptera: Apidae) infested by the mite *Varroa destructor* (Acari: Varroidae): a factor in colony loss during over-wintering? J Econ Entomol 2004 97 741 747 10.1603/0022-0493(2004)097[0741:APIWHB]2.0.CO;2 15279246

[B69] Corona M Libbrecht R Wurm Y Riba-Grognuz O Studer RA Keller L Vitellogenin underwent subfunctionalization to acquire caste and behavioral specific expression in the harvester ant *Pogonomyrmex barbatus* PLoS Genet 2013 9 8 e1003730 10.1371/journal.pgen.1003730 23966882 PMC3744404

[B70] Wegener J Huang ZY Lorenz MW Lorenz JI Bienefeld K New insights into the roles of juvenile hormone and ecdysteroids in honey bee reproduction J Insect Physiol 2013 59 7 655 661 10.1016/j.jinsphys.2013.04.006 23631954

[B71] Shpigler H Patch HM Cohen M Fan Y Grozinger CM Bloch G The transcription factor Kruppel homolog 1 is linked to hormone mediated social organization in bees BMC Evol Biol 2010 10 120 10.1186/1471-2148-10-120 20429952 PMC2876159

[B72] Van Doorn A Investigations into the regulation of dominance behavior and of the division of labor in bumblebee colonies *Bombus-terrestris* Netherl J Zool 1986 37 3–4 255 276

[B73] Alaux C Savarit F Jaisson P Hefetz A Does the queen win it all? Queen-worker conflict over male production in the bumblebee, *Bombus terrestris* Naturwissenschaften 2004 91 8 400 403 15278221 10.1007/s00114-004-0547-3

[B74] Sibbald ED Plowright CMS On the relationship between aggression and reproduction in pairs of orphaned worker bumblebees (*Bombus impatiens*) Insect Soc 2012 60 1 23 30

[B75] Amsalem E Shpigler H Bloch G Hefetz A Dufour’s gland secretion, sterility and foraging behavior: correlated behavior traits in bumblebee workers J Insect Physiol 2013 59 12 1250 1255 10.1016/j.jinsphys.2013.09.007 24100232

[B76] Lee YK Yoom HJ Lee SB Park IG Sohn HD Jin BR Molecular cloning and characterization of vitellogenin in *Bombus ignitus* J Ind Entomol 2009 18 1 33 40

[B77] Li J Huang J Cai W Zhao Z Peng W Wu J The vitellogenin of the bumblebee, Bombus hypocrita: studies on structural analysis of the cDNA and expression of the mRNA J Comp Physiol B 2010 180 2 161 170 10.1007/s00360-009-0434-5 20012056

[B78] Hornakova D Matouskova P Kindl J Valterova I Pichova I Selection of reference genes for real-time polymerase chain reaction analysis in tissues from *Bombus terrestris* and Bombus lucorum of different ages Analyt Biochem 2010 397 1 118 120 10.1016/j.ab.2009.09.019 19751695

[B79] Cameron SA Robinson GE Juvenile hormone does not affect division of labor in bumble bee colonies (Hymenoptera: Apidae) Ann Entomol Soc Am 1990 83 3 626 631

